# Polysubstance Use and Related Risk Behaviors among People Who Inject Drugs in Kenya Preparing for Hepatitis C Virus Treatment

**DOI:** 10.3390/v16081277

**Published:** 2024-08-10

**Authors:** Lindsey R. Riback, Mercy Nyakowa, John A. Lizcano, Chenshu Zhang, Peter Cherutich, Ann E. Kurth, Matthew J. Akiyama

**Affiliations:** 1 Department of Medicine, Albert Einstein College of Medicine, Montefiore Medical Center, Bronx, NY 10461, USAmatthew.akiyama@einsteinmed.edu (M.J.A.); 2 Kenya Ministry of Health, National AIDS&STI Control Program (NASCOP), 19361 Nairobi, Kenya; 3 Yale School of Nursing, Yale University, Orange, CT 06477, USA; 4 New York Academy of Medicine, New York, NY 10029, USA

**Keywords:** polysubstance use (PSU), Hepatitis C Virus (HCV), people who inject drugs (PWID), injection drug use (IDU), sub-Saharan Africa (SSA), low- and middle-income country (LMIC)

## Abstract

Polysubstance use (PSU), injection drug use (IDU), and equipment sharing are associated with bloodborne infection (BBI) transmission risk, particularly Hepatitis C Virus (HCV), yet data on PSU in low- and middle-income countries (LMICs) is limited. We report on baseline PSU, medication-assisted treatment (MAT) engagement, and motivation to reduce IDU among 95 people who inject drugs (PWID) who accessed needle and syringe programs (NSP) in Nairobi and Coastal Kenya prior to HCV treatment. Bivariate and multivariate logistic regression were used to examine the associations between PSU and behaviors that confer HCV transmission and acquisition risks. Most participants (70.5%) reported PSU in the last 30 days, and one-third (35.8%) reported PSU exclusive to just heroin and cannabis use. Common combinations were heroin and cannabis (49.3%), and heroin, cannabis, and bugizi (flunitrazepam) (29.9%). Participants at baseline were receiving MAT (69.5%), already stopped or reduced IDU (30.5%), and were HIV-positive (40%). PSU was significantly associated with IDU (*p* = 0.008) and the number of times (*p* = 0.016) and days (*p* = 0.007) injected in the last 30 days. Participants reported high PSU and equipment sharing, despite high MAT engagement. While co-locating BBI treatment within existing harm reduction services is necessary to promote uptake and curb re-infection, tailored services may be needed to address PSU, particularly in LMICs.

## 1. Introduction

Globally, there are an estimated 16 million people who inject drugs (PWID) [1]. PWID are at an increased risk of contracting Hepatitis C Virus (HCV), accounting for between 23% and 39% of new HCV infections and 10% of new Human Immunodeficiency Virus (HIV) infections globally [2], particularly in the absence of sterile injection equipment [3,4,5]. Ongoing injection drug use (IDU) after successful HCV treatment can also lead to HCV reinfection [6,7,8], among other adverse health impacts [7,9,10,11]. Therefore, it is important to understand substance use and injection practices among PWID preparing for HCV treatment, as this can aid in supporting and engaging individuals in appropriate harm reduction resources. Understanding such practices may be particularly important in resource-limited settings where financial barriers could limit retreatment options.

Recent findings suggest that the HCV antibody prevalence among PWID in sub-Saharan Africa (SSA) is 22% [1,12]. While this is lower than the global prevalence among PWID (52%) [1], access to direct-acting antivirals (DAA) remains limited in SSA [13], with only 1% of HCV-positive individuals in SSA having accessed HCV treatment [14]. Low rates of treatment access have been attributed to diminished access to medical care in general and the limited availability of HCV-related care such as DAA access and HCV providers [13].

While increasing DAA availability is critical to scale up, patient characteristics may influence treatment adherence and completion. Between 42% and 95% of PWID preparing for HCV treatment either report illicit substance use in the months leading up to treatment initiation or have a positive urine drug screening during this period [15,16,17,18,19], and more than 80% [18,20,21] report risk behaviors such as sharing injection equipment, which is concerning given the association between shared injection equipment, such as needles, cookers, and filters, with addiction severity [22,23]. Moreover, the act of front loading, using one syringe to prepare a substance solution and then dividing it among other syringes [24], has been significantly associated with HIV and HCV infection [25]. Polysubstance use (PSU)—consumption of more than one substance simultaneously or concurrently [26]—is also associated with addiction severity [27,28]. Recent findings suggest that PSU is not only associated with a higher prevalence of HCV [29] and HIV-related risk behaviors [30,31,32], but poorer physical and mental health outcomes as well [27]. However, these findings were obtained in the context of heroin and stimulants such as cocaine or methamphetamine [29,33], and mainly originate from high-income cohorts. Therefore, evidence from low- and middle-income countries (LMICs), particularly in SSA, remain scarce. 

The Government of Kenya has prioritized harm reduction services among PWID such as HCV treatment efforts in addition to existing medication-assisted treatment (MAT) and needle and syringe programs (NSP), the latter of which often serve as a referral source for MAT [34]. Despite these efforts, provider-level stigma and the presence of law enforcement in and around medical facilities have been identified as barriers to accessing HCV care for Kenyan PWID [35]. While recent modeling data suggests that scaling up DAAs alongside harm reduction interventions could reduce HCV incidence by 90% in Kenya by 2030 [36], clinicians’ concerns for the role that ongoing substance use may play in HCV transmission and reinfection remain [37]. Patients’ consumption behaviors and motivations for reducing IDU are important for providers to understand, as they may have implications for treatment and HCV transmission and acquisition, but it is also imperative that providers use these factors to connect patients with supportive services such as harm reduction programs, rather than allowing them to influence HCV treatment decisions. 

The goal of this sub-analysis is to examine rates of PSU and its association with behaviors that confer risk for the transmission and acquisition of bloodborne infections (BBIs) among PWID engaged in MAT and NSP in Kenya prior to HCV treatment. 

## 2. Materials and Methods

### 2.1. Study Population and Recruitment

This sub-analysis was part of a supplement to the Testing and Linkage to Care for Injection Drug Users (TLC-IDU) study (NCT01557998) [38]. In the supplement, we recruited participants intended to receive HCV treatment from 9 NSP service sites in Nairobi and coastal Kenya [39]. TLC-IDU participants for the parent study were 18 years old or older with a lifetime history of injecting drugs and reported use of any illicit non-prescribed drugs by any route of administration within the past 12 months [38,39]. The goal of the supplement was to determine HCV treatment outcomes and the correlates of sustained virologic response (SVR) among PWID engaged in MAT (specifically methadone) or NSPs [40]. Findings among the 92 who initiated treatment are described elsewhere [15,40].

In addition to completing a structured bio-behavioral survey assessing sociodemographic and behavioral risk factors [38,39], prospective participants were also tested for HCV with antibody (SD Bioline, Standard Diagnostics, South Korea) and HCV RNA testing (Abbott Molecular, Des Plaines, IL, USA) [39].

### 2.2. Procedures

Upon signing the consent form, all participants completed a bio-behavioral survey administered by a trained research assistant. Participants responded to questions regarding their demographics, risk behaviors related to HIV and HCV transmission, and services received at the NSP site. Participants were compensated 250 Kenyan shillings (USD 2.50) for their time and transportation. All participants were tested for HCV, HIV, and hepatitis B virus (HBV), and received a clinical evaluation and counseling on HCV and DAA therapy, as previously described [15,40]. 

### 2.3. Variables

We had two dependent variables of interest related to PSU. First, PSU was a binary variable in which any individual reporting heroin and another substance in the past 30 days was categorized as yes, regardless of the type or number of substances used. Hereafter, this variable is referred to as PSU. We also had a second binary variable for PSU, labeled PSU exclusive to just heroin and cannabis (PSU-EC), which included the use of heroin with substances other than or in addition to the use of cannabis. For example, a participant reporting the use of heroin and cannabis would be captured as reporting PSU under the first variable but not the second, PSU-EC. It is also important to note that in our cohort, all instances of PSU refer to the injection of heroin along with smoking, ingesting, or chewing other substances.

To gauge motivation to reduce IDU, participants were asked, “What do you think about reducing your injecting drug use, or stopping entirely?” Responses were captured as a categorical variable and then coded as a binary variable for use in the bivariate and multivariate analyses. As a categorical variable, responses were captured as follows: reducing or stopping for 6 months or more, reducing or stopping for the past 3–6 months, reducing or stopping within the past 3 months, ready to reduce or stop now, might be good but not ready, and no need to reduce or stop. This variable was collapsed into a binary variable, “Have already stopped or reduced IDU”, for the bivariate and multivariate analyses. Individuals reporting that they reduced their usage or stopped in the past 3 months, 3–6 months, or 6 or more months were coded as “yes” and all others were coded as “no”.

### 2.4. Statistical Analysis

We performed bivariate logistic regressions to examine the bivariate correlates of PSU and PSU-EC separately. We also conducted separate multivariate logistic regression models to estimate the likelihood of PSU and PSU-EC (odds ratios and 95% confidence intervals) associated with injection behaviors. The models incorporated the following independent variables hypothesized to be associated with both dependent variables: years injecting, IDU in the last 30 days, number of times injected in last 30 days, number of days injected in last 30 days, number of times using syringe before changing it, number of times using a syringe on a regular basis, and have already stopped or reduced IDU. In each multivariate logistic regression model, we also included variables with a maximum likelihood solution and *p* < 0.20 in the bivariate analyses as control variables. The adjusted odds ratios (AOR), 95% confidence intervals (CI), and *p* values were reported for the independent variables, where *p* values < 0.05 were considered to be statistically significant. We performed the above statistical analyses using SAS version 9.4.

## 3. Results

Between July 2017 and April 2018, 100 participants from the parent study were offered HCV treatment through the supplement. Of those offered, 95 accepted and enrolled in this supplement. Participant sociodemographic and clinical data are presented in Table 1. 

Participants were predominately male (85.3%) with a mean age of 36.5 years (standard deviation, SD ± 6.5). Regarding clinical characteristics, just over two-thirds (69.5%) were receiving MAT when offered treatment and less than half (40.0%) were HIV-positive. Most participants reported having ever been incarcerated (93.7%). Other demographic and clinical characteristics are reported in more detail elsewhere [15,40].

The majority of the cohort reported PSU (70.5%) in the last 30 days, most commonly heroin and cannabis (49.3%) followed by heroin, cannabis, and Bugizi (flunitrazepam) (29.9%), and heroin and Bugizi (flunitrazepam) (11.9%) among those reporting PSU. Roughly one-third of the cohort (35.8%) were classified as PSU-EC, representing just over half (50.7%) of those reporting PSU. 

Regarding substance use in general, almost all (94.7%) reported heroin use in the last 30 days. In the same timeframe, almost two-thirds (61.1%) reported smoking cannabis, 33.7% consumed Bugizi (flunitrazepam), and 9.5% khat. The majority (*n* = 87, 91.6%) of participants reported IDU on at least one day in the last 30 days.

Most reported that the NSP is their most importance source of syringes (89.5%) and that they used a needle an average of 1.3 (SD = ±1.3) times before changing it. Few reported that the needle they used was previously used by someone else (11.6%). Among these 11 individuals who used a needle after someone else had used it, only 6 individuals reported that the needle was used by someone they knew, although all were unaware of their injection partners’ HIV status. Most (78.9%) reported that they did not use any drug paraphernalia such as cookers or cotton after someone else during their last injection. 

Participants reported similar rates of passing along equipment to others as they did for receptive sharing. At their last injection, few reported passing their needle along to someone else (11.6%), and most (77.9%) did not pass along any drug paraphernalia to others. 

Regarding motivation to stop or reduce IDU, just under one-third (30.5%) reported they had already done so, of which 65.5% were on MAT. Among the 65.3% who reported that they were ready to stop or reduce their use, three-fourths (72.6%) were on MAT. Three individuals expressed that it might be good to make some changes, but were not quite ready. Only one participant indicated that they saw no need to stop or reduce their use despite reporting that they injected and smoked heroin, smoked cannabis, and chewed Bugizi (flunitrazepam) in the last 30 days. 

In the bivariate analyses, significant differences (*p* < 0.05) were noted related to PSU (Table 2a). For example, individuals reporting PSU were significantly more likely to report injecting at least once in the last 30 days compared with those who did not report PSU (97.0% vs. 78.6%; *p* = 0.008), injected more times on average (90.3 vs. 69.2; *p* = 0.039), and injected more days on average in the same timeframe (28.4 vs. 22.2; *p* = 0.024). While not significant, participants who reported PSU were less likely to report having already stopped or reduced their IDU (28.4% vs. 35.7%, *p* = 0.478).

Participants reporting PSU-EC were significantly less likely to live in Nairobi compared to those not reporting PSU-EC (2.9% vs. 19.7%; *p* = 0.028) and to have injected more times on average in the last 30 days (94.4 vs. 78.4; *p* = 0.052), although not significant (Table 2b). Individuals reported similar rates of having already stopped or reduced their IDU regardless of whether they reported PSU-EC or not (26.5% vs. 32.8%, *p* = 0.522).

We conducted separate multivariate regression analyses for each outcome of interest. The only variables that were significantly associated with PSU were related to injection, as follows: IDU in the last 30 days (OR = 12.6612, CI:1.9144–83.7368, *p* = 0.00845), number of times injected (OR = 1.0165, CI: 1.0031–1.0301, *p* = 0.01565), and number of days injected (OR = 1.0735, CI = 1.01945–1.1303, *p* = 0.00711) in the same timeframe (Table 3a). None of the independent variables were significantly associated with PSU-EC (*p* > 0.05) (Table 3b).

## 4. Discussion

In this study, we report on baseline substance use practices, including PSU, among PWID engaged in MAT and NSP in Kenya prior to HCV treatment initiation. To our knowledge, this is among the first studies to evaluate the association between PSU and behaviors that confer risk for BBI transmission among PWID in an LMIC. This study contributes to the dearth of information on PSU in resource-limited settings, particularly as they prepare for HCV treatment.

In the one study identified that reports on PSU among PWID in Kenya, Tun et al. found PSU to be common among PWID [41]. However, findings from this cohort did not compare factors associated with PSU, rather the prevalence of PSU, namely heroin in addition to other specific substances. Moreover, the earlier cohort [41] reported slightly higher rates of the following substance combinations when compared to our cohort: heroin and cannabis (66.5% vs. 60%), heroin and tranquilizer (50.1% vs. 33.7%), and heroin and khat (10.8% vs. 6.3%). While our cohort reports on PSU in two geographic areas, we do see lower rates of PSU in our cohort among the Nairobi participants; only one reported PSU of heroin and khat, and none reported the use of heroin and tranquilizer together. Other studies that characterize PSU among PWID in relation to HCV and HIV risk are often either confined to cohorts in high-income countries, specifically with heroin and stimulant use [29,33], or within the other populations in LMICs such as the general population [42] or female sex workers [43]; therefore, these findings provide unique insight into PWID in SSA. 

In our bivariate analyses, we found that participants who reported PSU-EC were significantly more likely to reside in coastal Kenya. This is somewhat expected given that coastal participants from the parent study were more likely to report engaging in risky behaviors including needle sharing, more times injecting on average in the last 30 days, and more years of injecting on average than those in Nairobi [39]. The higher rates of these behaviors, which are associated with greater addiction severity, have been attributed to the introduction of heroin to the Kenyan coast in the 1980s, which gradually spread inland over time [44]. In the only other study to assess the relationship between PSU and location in SSA, urban residence was found to be associated with PSU among a subset of male respondents who use drugs in the Ethiopia Demographic and Health Survey [45]. However, it is important to note that this finding was not specifically among PWID and only included males in the sample. 

While the reduction in IDU was not found to be statistically significant, we did identify high motivation to stop or reduce IDU, if not already reduced, among the overall cohort despite high rates of self-reported PSU and risky injection behaviors. This is of particular importance because studies examining the motivation to stop or reduce the use of illicit substances are mainly from higher-income settings which also include other substances such as stimulants [46,47]. For example, recent findings from a cohort of hospitalized PWID in Boston found a high motivation to reduce IDU despite not engaging in treatment [46]. Similarly, a cohort of PWID accessing NSPs in Appalachian Kentucky found that the majority of participants (69.9%) perceived a high importance in stopping or reducing their substance use, although their confidence to reduce was lower, with less than half (48.4%) reporting a high confidence to do so [47].

Regarding addiction severity, the high rate of ongoing or recent illicit substance use while preparing for HCV treatment mirrors the findings of other studies among individuals with opioid use disorder, where between 42% and 95% of participants preparing for HCV treatment report IDU [16,17,19]. Although we found comparable rates of recent illicit substance use, our cohort self-reported lower rates of injection equipment sharing, which could be attributed to the direct enrollment from NSPs. For example, recent surveys among Bangladeshi PWID found that 52–85% of respondents reported distributive and/or receptive syringe sharing in the last two months, and between 38 and 88% shared other injection paraphernalia in the same timeframe [18,20,21]. High rates of equipment sharing are indicative of addiction severity in other settings. A cohort of Canadian PWID with an addiction severity index (ASI) composite score of 0.4 or higher had higher odds of sharing cookers, water, filters, and swabs, and those who were HCV-positive had higher odds of sharing swabs [22,48]. Similarly, a cohort of British PWID found heroin dependence to be associated with increased rates of distributive sharing, particularly with their dealer [23]. While there is compelling evidence to suggest that there is no association between drug use during HCV treatment and adherence to direct DAAs [49] and/or achieving HCV cure [15,50,51,52], ongoing illicit substance use without harm reduction services increases the risk of reinfection post-treatment. 

It is important to note that while the association between PSU and high addiction severity is not unique to our cohort in a general sense, most studies that do examine PSU in the context of addiction severity are mainly from higher-income settings which also include other substances such as stimulants [29,33]. Moreover, those that do report on PSU do not compare risk factors between those engaging in PSU with those who do not [27,29,33]. While individuals in our cohort did not report injecting more than one substance, PSU does have important implications for HCV transmission, as recent findings have demonstrated injecting heroin, and at least one other substance has been associated with significantly higher HCV prevalence when compared with individuals who only inject heroin [29]. 

This study has some limitations. First, we enrolled PWID from NSP sites in Nairobi and coastal Kenya who were participants of the parent study and expressed interest in participating in this sub-study [15,40]. Given that we enrolled PWID who were already engaged in NSP services and some who were engaged in MAT, our participants may be more likely to seek treatment for substance use disorder and practice harm reduction, such as using clean needles at each injection and not sharing supplies, when compared to PWID who are not accessing these services. Additionally, our sample size may not have been adequately powered to detect differences for covariates (e.g., on MAT, HIV, sex, or criminal justice involvement). Another limitation is that, given the cross-sectional design of the study, we cannot ascertain the interrelationship of risk behaviors and PSU over time. Finally, substance use practices and motivation to stop or reduce IDU may not be representative of those for PWID in other SSA nations, particularly where harm reduction services are limited or nonexistent. 

## 5. Conclusions

In conclusion, despite high motivation to stop or reduce IDU and MAT engagement, our cohort reported high rates of PSU and some reported the recent sharing of injection equipment. This demonstrates a need for not only co-locating harm reduction services such as MAT and NSP to promote treatment uptake and reduce reinfection and risk for HCV and other BBIs, but also demonstrates a need for an emphasis on decentralized services that take a patient-centered approach by understanding and accounting for PSU and equipment-sharing behaviors among patients in LMICs.

## Figures and Tables

**Table 1:** Sociodemographic and clinical characteristics of participants (*n* = 95).

Age *	36.5 [±6.5]
Gender	
Male	81 (85.3%)
Female	14 (14.7%)
Location	
Nairobi	13 (13.7%)
Coast	82 (86.3%)
On MAT	66 (69.5%)
HIV-positive	38 (40.0%)
Ever held in jail	89 (93.7%)
**Injection behaviors**	
Age of first injection *	27.4 [±6.5]
Years injecting	8.9 [±5.9]
Inject on at least one day in the last 30 days	87 (91.6%)
Number of times injected in last 30 days *	84.1 [±38.6]
Number of days injected in last 30 days *	26.5 [±9.5]
Number of times using syringe before changing it *	1.3 [±1.3]
Which place is the most important source of syringes/needles for you?	
Places where I go to inject (the base, junkyard)	0 (0.0%)
Pharmacy	8 (8.4%)
Needle and syringe exchange	85 (89.5%)
Other IDUs	1 (1.1%)
Dealers	0 (0.0%)
Discarded trash	0 (0.0%)
Other	1 (1.1%)
How many other PWID do you know (by face or name)?	19.0 [±17.8]
What do you think about reducing your injecting drug use, or stopping entirely?	
Have already stopped or reduced	29 (30.5%)
Have been reducing or stopping for 6 months or more (*n* = 95)	6 (6.3%)
Have been reducing or stopping for the past 3–6 months (*n* = 95)	6 (6.3%)
Have been reducing or stopping within the past 3 months (*n* = 95)	17 (17.9%)
Have not yet stopped or reduced	66 (69.5%)
Ready to reduce or stop now (*n* = 95)	62 (65.3%)
Might be good, but not ready (*n* = 95)	3 (3.2%)
No need to reduce or stop (*n* = 95)	1 (1.1%)
**Equipment sharing behaviors**	
Ever backloaded/frontloaded	20 (21.1%)
Needle/syringe used at last injection was previously used by someone else	11 (11.6%)
By someone I know (*n* = 11)	6 (54.5%)
Who I knew was HIV-positive (*n* = 6)	0 (0.0%)
Who I knew was HIV-negative (*n* = 6)	0 (0.0%)
Whose HIV status I did not know (*n* = 6)	6 (100.0%)
By someone I did not know	5 (45.5%)
Used equipment previously used by someone else at last injection	
Cookers	16 (16.8%)
Cotton	12 (12.6%)
Water/Solution	20 (21.1%)
Bleach	11 (11.6%)
Nothing was previously used	75 (78.9%)
Passed along needle/syringe to someone else to use afterwards at last injection	11 (11.6%)
Passed along equipment to someone else to use afterwards at last injection	
Cookers	16 (16.8%)
Cotton	13 (13.7%)
Water/Solution	19 (20.0%)
Bleach	11 (11.6%)
Nothing was passed on	74 (77.9%)
**Substance-specific behaviors in the last 30 days**	
Heroin	90 (94.7%)
Injected	87 (96.7%)
Smoked	23 (25.6%)
Cannabis	58 (61.1%)
Khat	9 (9.5%)
Bugizi (flunitrazepam)	32 (33.7%)
Chewed	18 (56.3%)
Ingested	14 (56.3%)
Polysubstance use (PSU)	67 (70.5%)
Heroin and Cannabis	33 (49.3%)
Heroin and Khat	2 (3.0%)
Heroin and Bugizi (flunitrazepam)	8 (11.9%)
Heroin, Bugizi (flunitrazepam) and Cannabis	20 (29.9%)
Heroin, Bugizi (flunitrazepam), Cannabis, and Khat	4 (6.0%)
Polysubstance exclusive of just heroin and cannabis (PSU-EC)	34 (35.8%)

Note: * mean, standard deviation.

**Table 2:** Covariates of injection behaviors on polysubstance use among participants.

2a. Polysubstance Use			
	Polysubstance Use (*n* = 67, 70.5%)	No Polysubstance Use (*n* = 28, 29.5%)	*p*-Value
Age *	36.5 [±6.3]	36.4 [±7.2]	0.954
Male	59 (88.1%)	22 (78.6%)	0.234
Nairobi ^	7 (10.5%)	6 (21.4%)	0.156
On MAT	45 (67.2%)	21 (75.0%)	0.45
Ever held in jail	63 (94.0%)	26 (92.9%)	0.83
Age of first injection *	27.8 [±5.9]	26.6 [±7.7]	0.448
Years injecting	8.6 [±5.5]	9.5 [±6.9]	0.521
Inject in the last 30 days	65 (97.0%)	22 (78.6%)	0.008
Number of times injected in last 30 days *	90.3 [±32.6]	69.2 [±47.7]	0.039
Number of days injected in last 30 days *	28.4 [±6.8]	22.2 [±13.1]	0.024
Number of times using syringe before changing it *	1.2 [±1.1]	1.6 [±1.7]	0.356
How many other PWID do you know (by face or name)?	17.8 [±14.3]	21.8 [±24.3]	0.419
What do you think about reducing your injecting drug use, or stopping entirely?			
Have already stopped or reduced	19 (28.4%)	10 (35.7%)	0.478
Have not yet stopped or reduced	48 (71.6%)	18 (64.3%)	
Needle/syringe used at last injection was previously used by someone else	9 (13.4%)	2 (7.1%)	0.382
HIV-positive	27 (40.3%)	11 (39.3)	0.927
**2b. Polysubstance exclusive of just heroin and cannabis (PSU-EC)**			
	Polysubstance use (*n* = 34, 35.8%)	No polysubstance use (*n* = 61, 62.4%)	*p*-value
Age *	35.7 [±6.1]	36.9 [±6.8]	0.408
Male	28 (82.4%)	53 (86.9%)	0.55
Nairobi ^	1 (2.9%)	12 (19.7%)	0.028
On MAT	21 (61.8%)	45 (73.8%)	0.223
Ever held in jail	33 (97.1%)	56 (91.8%)	0.415
Age of first injection *	27.4 [±5.7]	27.5 [±6.9]	0.95
Years injecting	8.3 [±5.4]	9.2 [±6.2]	0.44
Inject in the last 30 days	33 (97.1%)	54 (88.5%)	0.252
Number of times injected in last 30 days *	94.4 [±34.0]	78.4 [±40.1]	0.052
Number of days injected in last 30 days *	28.3 [±6.8]	25.6 [±10.6]	0.126
Number of times using syringe before changing it *	1.4 [±1.6]	1.3 [±1.2]	0.687
How many other PWID do you know (by face or name)?	17.7 [±16.8]	19.7 [±18.4]	0.602
What do you think about reducing your injecting drug use, or stopping entirely?			
Have already stopped or reduced	9 (26.5%)	20 (32.8%)	0.522
Have not yet stopped or reduced	25 (73.5%)	41 (67.2%)	
Needle/syringe used at last injection was previously used by someone else	5 (14.7%)	6 (9.8%)	0.477
HIV-positive	15 (44.1%)	23 (37.7)	0.541

Note: * mean, standard deviation. ^ Nairobi as the reference variable.

**Table 3:** Effects of injection behaviors on polysubstance use in multivariate logistic regression.

3a. Polysubstance Use		
	OR (95% CI)	*p* Value
Years injecting *	0.77 [0.46–1.29]	0.313
Inject in the last 30 days	12.67 [1.91–83.74]	0.008
Number of times injected in last 30 days *	1.02 [1.00–1.03]	0.016
Number of days injected in last 30 days *	1.07 [1.02–1.13]	0.007
Number of times using syringe before changing it *	0.84 [0.58–1.22]	0.361
On a regular basis, how many times do you use a syringe? *	0.91 [0.65–1.27]	0.570
Has reduced injection drug use	0.70 [0.23–2.09]	0.519
**3b. Polysubstance exclusive of just heroin and cannabis (PSU-EC)**		
Years injecting *	1.04 [0.54–2.01]	0.896
Inject in the last 30 days	4.27 [0.40–45.67]	0.230
Number of times injected in last 30 days *	1.01 [1.00–1.03]	0.076
Number of days injected in last 30 days *	1.04 [0.98–1.10]	0.224
Number of times using syringe before changing it *	1.18 [0.82–1.70]	0.370
On a regular basis, how many times do you use a syringe? *	1.27 [0.90–1.80]	0.181
Has reduced injection drug use	0.51 [0.17–1.52]	0.228

Note: These are separate multivariate analyses; each analysis has been controlled for age, gender, HIV status, MAT status, location, lifetime history of incarceration, age of first injection, number of other known PWID, and having used a syringe at last injection that was previously used by someone else in order to control for collinearity. OR= odds ratio; CI= confidence interval; * For continuous variables, ORs are for per unit increase in that variable.

## Data Availability

The data presented in this study are available on request from the corresponding author due to privacy and ethical restrictions.
